# Trends and Regional Variation in Prevalence of Cardiovascular Risk Factors and Association With Socioeconomic Status in Canada, 2005-2016

**DOI:** 10.1001/jamanetworkopen.2021.21443

**Published:** 2021-08-19

**Authors:** Haijiang Dai, Arwa Younis, Jude Dzevela Kong, Nicola Luigi Bragazzi, Jianhong Wu

**Affiliations:** 1 Laboratory for Industrial and Applied Mathematics, Centre for Disease Modelling, York University, Toronto, Ontario, Canada; 2 Clinical Cardiovascular Research Center, University of Rochester Medical Center, Rochester, New York

## Abstract

**Importance:**

Cardiovascular disease remains the second leading cause of death in Canada. Monitoring and tracking the trends and disparities in major cardiovascular risk factors could provide benchmarks for future cardiovascular health strategies.

**Objective:**

To investigate the temporal trends, regional variations, and socioeconomic disparities in major cardiovascular risk factors in Canada from 2005 to 2016.

**Design, Setting, and Participants:**

This repeated cross-sectional survey study included adults aged 20 years and older from 6 Canadian Community Health Survey cycles between 2005 and 2016. Cardiovascular risk factors included hypertension, diabetes, obesity, and current smoking. Socioeconomic status was measured using equivalized household income. Data analysis was performed from September 2019 to April 2020.

**Exposures:**

A total of 112 health regions and socioeconomic status.

**Main Outcomes and Measures:**

Age- and sex-adjusted prevalence of hypertension, diabetes, obesity, and current smoking by year; health regions; and socioeconomic status. Absolute numbers were rounded to base 100 for confidentiality purposes, and percentages were based on weighted numbers. Slope index of inequality (SII) and relative index of inequality (RII) were calculated to assess absolute and relative socioeconomic inequalities, respectively.

**Results:**

A total of 670 000 respondents (329 000 [49.1%] men; 341 000 [50.9%] women) aged 20 years and older from 6 survey cycles were enrolled for this study. The largest age group was those aged 40 to 59 years (eg, 2005 cycle: 40.2% [95% CI, 39.9%-40.6%]). In the 2015/2016 cycle, the overall age- and sex-adjusted prevalence rates of hypertension, diabetes, obesity, and current smoking were 20.7% (95% CI, 20.4%-21.1%), 7.2% (95% CI, 7.0%-7.5%), 20.1% (95% CI, 19.7%-20.6%), and 17.8% (95% CI, 17.4%-18.2%), respectively. From 2005 to 2016, there was a significant increase in the prevalence of hypertension, diabetes, and obesity (eg, prevalence of diabetes in both sexes, 2005: 5.8% [95% CI, 5.6%-6.0%]; 2015/2016: 7.2% [95% CI, 7.0%-7.5%]; *P* < .001) but a significant decrease in the prevalence of current smoking (both sexes, 2005: 22.1% [95% CI, 21.7%-22.5%]; 2015/2016: 17.8% [95% CI, 17.4%-18.2%]; *P* < .001). The prevalence of all the risk factors varied widely across health regions (eg, obesity, Vancouver Health Service Delivery Area: 6.7% [95% CI, 4.5%-9.0%]; Miramichi Area: 36.8% [95% CI, 27.3%-46.3%]). In addition to obesity among men, all risk factors tended to be more common among those with lower income (eg, prevalence of hypertension in both sexes, 2015/2016, lowest income group: 23.2% [95% CI, 22.4%-24.0%]; highest income group: 18.4% [95% CI, 17.7%-19.1%]). The SII and RII indicated consistent absolute and relative socioeconomic inequalities in hypertension, diabetes, and current smoking over time (eg, RII for hypertension in both sexes, 2005: 1.25; 95% CI, 1.18-1.33; 2015/2016: 1.34; 95% CI, 1.26-1.43). However, the phenomenon of absolute and relative socioeconomic inequalities in obesity was only observed among women (eg, RII for 2015/2016 for obesity in women; 1.74 (95% CI, 1.56-1.93); men: 1.09; 95% CI, 0.99-1.21).

**Conclusions and Relevance:**

During the study period, the prevalence of hypertension, diabetes, and obesity significantly increased, while the prevalence of current smoking significantly decreased. Geographic and socioeconomic gaps should be considered and addressed in future interventions and policies targeted at reducing these cardiovascular risk factors in Canada.

## Key Points

**Question:**

What are the trends and patterns of regional and socioeconomic disparities in cardiovascular risk factors in Canada from 2005 to 2016?

**Findings:**

This survey study of 670 000 adults found a significant increase in the prevalence of hypertension, diabetes, and obesity but a significant decrease in the prevalence of current smoking, with prevalence rates varying widely across health regions. In addition to obesity among men, persistent absolute and relative socioeconomic inequalities were observed for all risk factors.

**Meaning:**

These findings suggest that geographic and socioeconomic gaps should be considered and addressed in future cardiovascular health strategies in Canada.

## Teaser

This survey study investigates the temporal trends, regional variations, and socioeconomic disparities in major cardiovascular risk factors in Canada from 2005 to 2016.

## Introduction

Cardiovascular disease remains the second leading cause of death in Canada and has imposed a huge economic burden on Canadian health care systems.^1,2^ Convincing evidence demonstrates that management of major risk factors, such as hypertension, diabetes, obesity, and smoking, could effectively reduce cardiovascular events and mortality.^3,4,5^ Unfortunately, besides smoking, a continuing increase in the prevalence of these cardiovascular risk factors has been reported in the Canadian population between 1994 and 2005.^6^ Moreover, regional variations in the prevalence and treatment of these risk factors make this situation even more complicated.^7^ To reverse these unfavorable trends, several prevention strategies, such as the Cardiovascular Health Awareness Program and Canadian Hypertension Education program, have been launched and widely implemented.^8,9^ However, the lack of nationally representative data investigating the latest trends in hypertension, diabetes, obesity, and smoking makes it difficult to assess the impact of prevention programs on the Canadian population. Additionally, recent studies suggest that in Canada, socioeconomic inequalities in health may be widening over time.^10,11^ Monitoring and tracking socioeconomic disparities in hypertension, diabetes, obesity, and smoking could provide benchmarks for future health strategies; however, less is known about the secular trends in socioeconomic inequalities with regard to these major cardiovascular risk factors in Canada. Thus, the aims of this study were to provide an update of the secular trends in the prevalence of hypertension, diabetes, obesity, and smoking at the national and regional levels and to estimate relative and absolute socioeconomic inequalities in these major cardiovascular risk factors in Canada between 2005 and 2016.

## Methods

### Data Sources

The Canadian Community Health Survey (CCHS), conducted by Statistics Canada, has been a nationally representative cross-sectional survey study since 2000. Details about the design and conduct of the CCHS are available online.^12^ In brief, using consistent, multistage, stratified cluster sampling strategies, a nationally representative sample was selected for each survey in 1-year or 2-year cycles. The survey excluded people living on Indian reserves or Crown lands, people living in institutions, full-time members of the Canadian military, and residents of certain remote regions (all representing <3% of the population). For the current analysis, we included adults aged 20 years and older from the latest 6 cycles (done in 2005, 2007/2008, 2009/2010, 2011/2012, 2013/2014, and 2015/2016) of the CCHS. Ethical approval of the CCHS was obtained from the relevance policy committees at Statistics Canada, and all respondents provided written informed consent. Per Statistics Canada, additional institutional review board approval for this study was not needed. This study followed the American Association for Public Opinion Research (AAPOR) reporting guideline.

### Measures

Our study focused on 4 major cardiovascular risk factors: hypertension, diabetes, obesity, and current smoking. For all surveys, the ascertainment of hypertension was based on questions about whether a respondent recalled receiving a physician diagnosis of hypertension or taking medications for hypertension.^13^ Similarly, diabetes was determined by questions about whether a respondent recalled receiving a physician diagnosis of diabetes or taking medications for diabetes. Obesity was defined as a body mass index (BMI; calculated as weight in kilograms divided by height in meters squared) of 30 or greater according to clinical guidelines^14^; BMI was calculated using self-reported height and weight. Women who were pregnant during the survey were excluded from the analysis of obesity. Current smoking was defined as now smoking cigarettes occasionally or daily and having smoked more than 100 cigarettes in lifetime.^15^

Health regions refer to administrative areas divided by Canadian provincial government to administer and deliver health care services to all residents. Because the boundaries change over time,^16^ health regions were combined to ensure stable units of analysis over the period 2005 to 2016, reducing the number of areas analyzed from 112 to 105 (eTable 1 in the Supplement). Additionally, as suggested by Statistics Canada,^16^ 3 neighboring health regions in Saskatchewan, which have small populations, were combined to avoid potential poor data quality (eTable 2 in the Supplement). For simplicity, combined areas are referred to as health regions throughout.

Socioeconomic status was measured using equivalized household income, which is defined as total annual household income divided by the square root of household size.^17,18^ To ensure a consistent comparison over the period 2005 to 2016, equivalized household income was then categorized into quartiles in each survey cycle.

### Statistical Analysis

Statistical analyses were conducted using Stata version 15.0 (StataCorp) and R version 3.6.1 (R Project for Statistical Computing). Statistical significance was met with 2-tailed *P* < .05. For all surveys, sampling weights provided by Statistics Canada were applied to obtain population estimates. Bootstrap methods provided by Statistics Canada were used to account for complex survey design. Absolute numbers were rounded to base 100 for confidentiality purposes according to Statistics Canada data release policies. Percentages were based on weighted numbers. The age- and sex-adjusted prevalence of cardiovascular risk factors was calculated by direct standardization to the 2006 Canada Census population using the joint age (10-year interval) and sex groups. Temporal trends in prevalence of cardiovascular risk factors were evaluated by multivariable Poisson regression analysis, adjusting for age and sex.^19,20^ *P* values for trend were then measured using the contrast postestimation command in Stata.

We examined absolute socioeconomic inequalities in cardiovascular risk factors with the slope index of inequality (SII) and relative socioeconomic inequalities with the relative index of inequality (RII).^21,22,23^ To calculate the SII and RII, we first transformed socioeconomic status into cumulative rank probabilities (ridit scores) ranging from 0 (highest) to 1 (lowest).^22^ Then, we used multivariable Poisson regression models, adjusting for age and sex, to estimate the association between the ridit scores and prevalence of cardiovascular risk factors. The RII was derived from the regression models, and the SII was obtained using marginal effects and the nlcom postestimation commands in Stata. Temporal trends in the SII and RII were assessed by adding the interaction term between the ridit scores and survey circles into the regression models.^24^ *P* values for trend were then calculated using the contrast postestimation command in Stata.

## Results

A total of 670 000 respondents (329 000 [49.1%] men; 341 000 [50.9%] women) aged 20 years and older from 6 survey cycles were enrolled for this study. The largest age group were those aged 40 to 59 years (eg, 2005 cycle: 40.2% [95% CI, 39.9%-40.6%]). The sample size and the distribution of study sample by sex and age group in each survey are shown in Table 1. The percentage of missing values for each cardiovascular risk factor (ie, hypertension, diabetes, obesity, and current smoking) is also shown in Table 1. Respondents who had missing values were excluded from the respective analysis of each cardiovascular risk factor.

**Table 1.** Baseline Characteristics of Study Sample

Characteristic	Respondents, % (95% CI)					
	2005	2007/2008	2009/2010	2011/2012	2013/2014	2015/2016
Sample size^a^	116 600	117 400	110 300	111 800	115 000	98 900
Weighted population^a^	23 778 300	24 651 000	25 380 400	26 098 300	26 833 900	27 462 100
Sex						
Men	49.0 (49.0-49.0)	49.0 (49.0-49.1)	49.1 (49.1-49.1)	49.1 (49.1-49.1)	49.1 (49.1-49.1)	49.1 (49.0-49.2)
Women	51.0 (51.0-51.0)	51.0 (51.0-51.0)	50.9 (50.9-50.9)	50.9 (50.9-50.9)	50.9 (50.9-50.9)	50.9 (50.8-51.0)
Age group, y						
20-39	36.6 (36.4-36.9)	36.2 (36.0-36.5)	35.3 (35.0-35.6)	35.3 (35.0-35.6)	35.1 (34.8-35.4)	34.6 (34.3-34.9)
40-59	40.2 (39.9-40.6)	39.9 (39.5-40.3)	39.7 (39.2-40.2)	38.1 (37.6-38.6)	37.4 (36.9-37.9)	36.6 (36.2-37.0)
60-79	19.5 (19.3-19.7)	20.1 (19.8-20.4)	21.0 (20.7-21.4)	22.4 (22.0-22.8)	23.3 (22.9-23.7)	24.6 (24.3-24.9)
≥80	3.7 (3.6-3.8)	3.8 (3.7-3.9)	4.0 (3.9-4.1)	4.2 (4.0-4.3)	4.2 (4.1-4.4)	4.2 (4.0-4.4)
Missing values^b^						
Hypertension	0.20 (0.16-0.25)	0.30 (0.26-0.35)	0.23 (0.19-0.28)	0.28 (0.23-0.34)	0.27 (0.22-0.33)	0.44 (0.38-0.52)
Diabetes	0.09 (0.06-0.12)	0.11 (0.08-0.14)	0.05 (0.04-0.07)	0.10 (0.08-0.14)	0.12 (0.09-0.17)	0.14 (0.10-0.19)
Obesity	3.20 (3.05-3.36)	5.44 (5.22-5.67)	5.17 (4.96-5.39)	5.15 (4.93-5.38)	5.31 (5.08-5.56)	6.54 (6.28-6.81)
Current smoking	0.54 (0.44-0.66)	0.27 (0.22-0.31)	0.29 (0.25-0.35)	0.38 (0.32-0.45)	0.38 (0.33-0.45)	0.29 (0.25-0.35)

^a^ Numbers are rounded to base 100 for confidentiality purposes according to Statistics Canada data release policies. Percentages are based on weighted numbers.

^b^ Participants who responded “Don’t know,” “Refusal,” or “Not stated” to the questions were regarded as having missing values and thus not included in respective analysis. For obesity, participants with missing values included women who are pregnant.

### Prevalence of Cardiovascular Risk Factors in 2015/2016 Cycle

The age- and sex-adjusted prevalence of each cardiovascular risk factor in 2015/2016 overall and by sex and age group was shown in Table 2 and eTable 3 in the Supplement. The overall age- and sex-adjusted prevalence of hypertension, diabetes, obesity, and current smoking was 20.7% (95% CI, 20.4%-21.1%), 7.2% (95% CI, 7.0%-7.5%), 20.1% (95% CI, 19.7%-20.6%), and 17.8% (95% CI, 17.4%-18.2%), respectively; all risk factors tended to be higher among men. Additionally, as expected, the adjusted prevalence of hypertension and diabetes increased substantially with age. By contrast, the adjusted prevalence of obesity and current smoking was lowest in those aged 80 years or older. Notably, 14.8% (95% CI, 14.4%-15.1%) of the respondents reported an accumulation of at least 2 cardiovascular risk factors.

**Table 2.** Age- and Sex-Adjusted Prevalence of Cardiovascular Risk Factors, Overall and by Sex

Risk factor	Respondents, % (95% CI)						*P* value	Direction of trend
	2005	2007/2008	2009/2010	2011/2012	2013/2014	2015/2016		
**Both sexes**								
Hypertension	19.1 (18.8-19.4)	20.0 (19.7-20.3)	20.3 (19.9-20.7)	20.3 (19.9-20.6)	20.1 (19.7-20.4)	20.7 (20.4-21.1)	<.001	Increase
Diabetes	5.8 (5.6-6.0)	6.7 (6.5-7.0)	6.9 (6.7-7.2)	6.9 (6.6-7.1)	7.0 (6.8-7.3)	7.2 (7.0-7.5)	<.001	Increase
Obesity	16.1 (15.8-16.5)	17.3 (16.9-17.6)	18.2 (17.8-18.6)	18.6 (18.1-19.0)	19.7 (19.2-20.1)	20.1 (19.7-20.6)	<.001	Increase
Current smoking	22.1 (21.7-22.5)	22.1 (21.7-22.5)	20.8 (20.4-21.3)	20.7 (20.2-21.2)	19.2 (18.8-19.7)	17.8 (17.4-18.2)	<.001	Decrease
≥2 Risk factors	12.5 (12.2-12.8)	13.7 (13.4-14.0)	14.2 (13.9-14.6)	14.3 (13.9-14.6)	14.4 (14.0-14.7)	14.8 (14.4-15.1)	<.001	Increase
**Men** ^**a**^								
Hypertension	18.8 (18.3-19.2)	19.9 (19.5-20.4)	20.6 (20.0-21.1)	20.9 (20.4-21.4)	21.6 (21.0-22.1)	22.1 (21.5-22.6)	<.001	Increase
Diabetes	6.6 (6.3-6.9)	7.6 (7.3-8.0)	8.2 (7.8-8.6)	7.7 (7.3-8.0)	8.1 (7.7-8.5)	8.3 (7.9-8.6)	<.001	Increase
Obesity	17.2 (16.6-17.7)	18.3 (17.8-18.8)	19.6 (19.0-20.2)	19.6 (19.0-20.2)	21.2 (20.5-21.9)	21.6 (21.0-22.3)	<.001	Increase
Current smoking	24.1 (23.6-24.7)	24.8 (24.2-25.4)	23.7 (23.0-24.3)	23.4 (22.7-24.1)	22.4 (21.7-23.1)	20.4 (19.8-21.0)	<.001	Decrease
≥2 Risk factors	13.2 (12.8-13.6)	14.7 (14.3-15.2)	15.7 (15.1-16.2)	15.5 (14.9-16.1)	16.4 (15.8-17.0)	16.7 (16.2-17.3)	<.001	Increase
**Women** ^**a**^								
Hypertension	19.3 (18.9-19.7)	19.9 (19.5-20.4)	19.8 (19.4-20.3)	19.6 (19.1-20.1)	18.6 (18.1-19.0)	19.3 (18.8-19.8)	.015	Decrease
Diabetes	5.1 (4.9-5.3)	5.9 (5.7-6.2)	5.8 (5.6-6.1)	6.2 (5.9-6.6)	6.1 (5.8-6.4)	6.3 (6.0-6.7)	<.001	Increase
Obesity	15.1 (14.6-15.5)	16.2 (15.8-16.7)	16.8 (16.3-17.3)	17.5 (17.0-18.1)	18.2 (17.6-18.7)	18.7 (18.1-19.3)	<.001	Increase
Current smoking	20.1 (19.6-20.6)	19.5 (19.0-20.0)	18.1 (17.6-18.6)	18.2 (17.6-18.8)	16.1 (15.6-16.7)	15.3 (14.7-15.8)	<.001	Decrease
≥2 risk factors	11.8 (11.5-12.2)	12.6 (12.2-13.0)	12.8 (12.4-13.3)	13.1 (12.6-13.5)	12.5 (12.1-12.9)	12.9 (12.4-13.4)	.005	Increase

^a^ Sex comparisons are only adjusted for age.

### Trend Analyses

From 2005 to 2016, there was a statistically significant increase in the overall age- and sex-adjusted prevalence of hypertension, diabetes, and obesity (eg, prevalence of diabetes in both sexes, 2005: 5.8% [95% CI, 5.6%-6.0%]; 2015/2016: 7.2% [95% CI, 7.0%-7.5%]; *P* < .001), but a statistically significant decrease in the overall age- and sex-adjusted prevalence of current smoking (both sexes, 2005: 22.1% [95% CI, 21.7%-22.5%]; 2015/2016: 17.8% [95% CI, 17.4%-18.2%]; *P* < .001). Temporal trends in the age- and sex-adjusted prevalence of cardiovascular risk factors by sex and age group are shown in Table 2 and eTable 3 in the Supplement. Between 2005 and 2016, the adjusted prevalence of hypertension significantly increased among men aged 40 to 59 years, 60 to 79 years, and 80 years and older as well as among women aged 80 years and older, but it slightly decreased among women aged 40 to 59 years and 60 to 79 years; the adjusted prevalence of diabetes significantly increased among men aged 40 to 59 years, 60 to 79 years, and 80 years and older as well as among women in the same age groups; the adjusted prevalence of obesity significantly increased among men in all age groups and among women aged 20 to 39 years, 40 to 59 years, and 60 to 79 years; and the adjusted prevalence of current smoking significantly declined among men aged 20 to 39 years, 40 to 59 years, and 80 years and older as well as among women in all age groups (eTable 3 in the Supplement).

### Regional Variations

**Figure 1.**
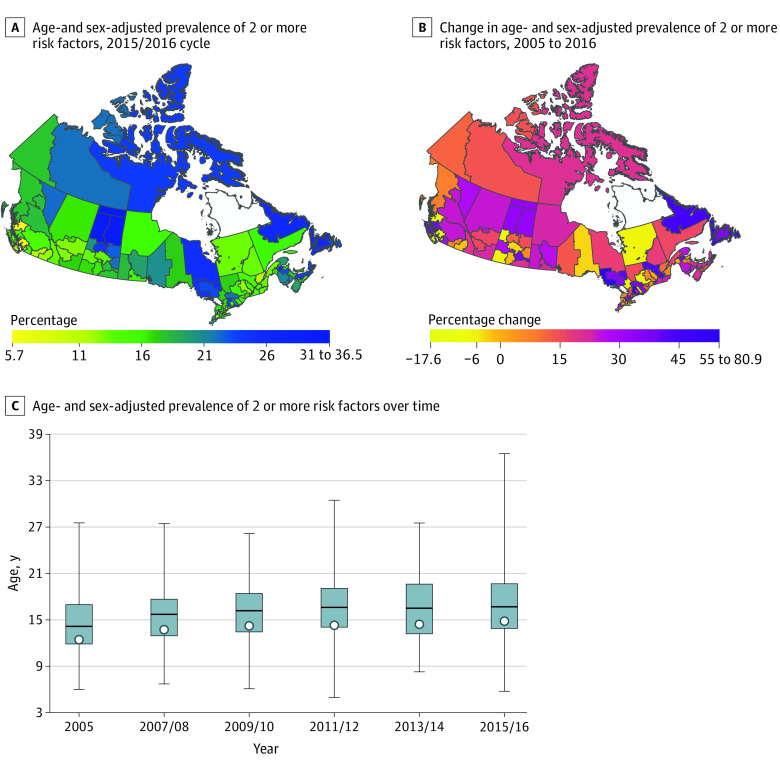
Health Region–Level Age- and Sex-Adjusted Prevalence of 2 or More Cardiovascular Risk Factors A and B, 2 health regions (ie, Région du Nunavik and Région des Terres Cries de la Baie James) are blank because of missing data. C, The bottom border, middle line, and top border of the boxes indicate the 25th, 50th, and 75th percentiles, respectively, across all health regions; the whiskers indicate the full range across all health regions; and the circles indicate the national-level prevalence rate.

In 2015/2016, the age- and sex-adjusted prevalence of hypertension, diabetes, obesity, current smoking (eFigures 1-4 in the Supplement), and 2 or more cardiovascular risk factors (Figure 1) varied widely across health regions. For hypertension, the health region–level age- and sex-adjusted prevalence ranged from 12.7% (95% CI, 10.6%-14.9%) in the North Shore/Coast Garibaldi Health Service Delivery Area to 31.0% (95% CI, 23.2%-38.7%) in the Mamawetan/Keewatin/Athabasca Regional Health Authorities; for diabetes, it ranged from 3.9% (95% CI, 2.9%-4.8%) in the Okanagan Health Service Delivery Area to 15.3% (95% CI, 8.7%-21.9%) in the Mamawetan/Keewatin/Athabasca Regional Health Authorities; for obesity, it ranged from 6.7% (95% CI, 4.5%-9.0%) in the Vancouver Health Service Delivery Area to 36.8% (95% CI, 27.3%-46.3%) in the Miramichi Area; for current smoking, it ranged from 11.0% (95% CI, 7.8%-14.2%) in the Richmond Health Service Delivery Area to 62.4% (95% CI, 57.4%-67.4%) in Nunavut; and for 2 or more cardiovascular risk factors, it ranged from 5.7% (95% CI, 3.8%-7.6%) in the Eastern Regional Health Authority to 36.5% (95% CI, 28.0%-45.0%) in the Mamawetan/Keewatin/Athabasca Regional Health Authorities.

Although national age- and sex-adjusted prevalence of hypertension, diabetes, obesity, and 2 or more cardiovascular risk factors increased between 2005 and 2016 (Table 2), there were 26, 24, 10, and 19 health regions, respectively, that experienced a decline in the prevalence rates over the same period. Conversely, although national age- and sex-adjusted prevalence of current smoking declined between 2005 and 2016 (Table 2), 9 health regions experienced an increase over this period (eg, Campbellton area: 2005, 20.9% [95% CI, 14.9%-26.9%]; 2015/2016, 39.0% [95% CI, 28.7%-49.3%]; 86.9% increase; Nunavut: 2005, 46.0% [95% CI, 39.5%-52.9%]; 2015/2016, 62.4% [95% CI, 57.4%-67.4%]; 35.5% increase). Detailed information about health regions with the most rapid increase or decrease in age- and sex-adjusted prevalence of each cardiovascular risk factor is shown in eTable 4 in the Supplement.

### Socioeconomic Inequalities

**Figure 2.**
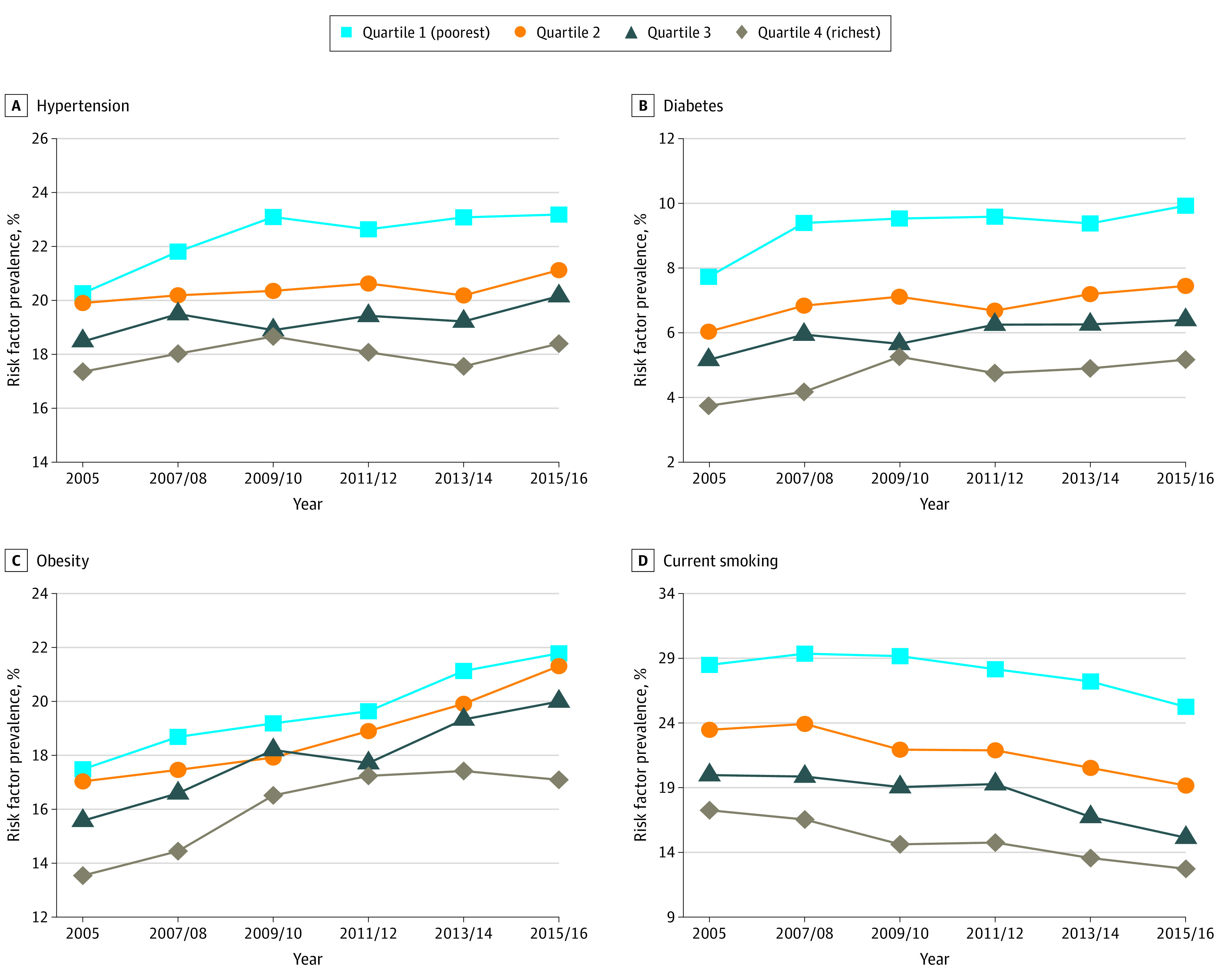
Overall Age- and Sex-Adjusted Prevalence of Cardiovascular Risk Factors by Income Quartile

The distribution of age- and sex-adjusted prevalence of each cardiovascular risk factor across income quartiles overall and by sex is presented in Figure 2 and eFigure 5 in the Supplement. In addition to obesity among men, all cardiovascular risk factors tended to be more common among those with lower income (eg, prevalence of hypertension in both sexes, 2015/2016, quartile 1: 23.2% [95% CI, 22.4%-24.0%]; quartile 4: 18.4% [95% CI, 17.7%-19.1%]; prevalence of hypertension in men, 2015/2016, quartile 1: 24.0% [95% CI, 22.7%-25.4%]; quartile 4: 20.6% [95% CI, 19.6%-21.6%]; prevalence of hypertension in women, 2015/2016, quartile 1: 22.2% [95% CI, 21.2%-23.2%]; quartile 4: 16.2% [95% CI, 15.2%-17.1%]). The SII and RII presented in Table 3 suggested consistent absolute and relative socioeconomic inequalities in hypertension, diabetes, and current smoking over time (eg, RII for hypertension in both sexes, 2005: 1.25 [95% CI, 1.18-1.33]; 2015/2016: 1.34 [95% CI, 1.26-1.43]). However, the phenomenon of absolute and relative socioeconomic inequalities in obesity was only found among women (eg, RII for 2015/2016 for obesity in women; 1.74 [95% CI, 1.56-1.93]; men: 1.09 [95% CI, 0.99-1.21]). Trend analyses showed that there was a statistically significant increase in the SII and RII for hypertension and a statistically significant increase in the RII for current smoking (Table 3), suggesting the corresponding absolute and relative socioeconomic inequalities were widening between 2005 and 2016.

**Table 3.** Changes in Absolute and Relative Socioeconomic Inequalities for Each Cardiovascular Risk Factor

Year	Predicted mean (95% CI)										
	Both sexes^a^				Men^b^				Women^b^		
	SII		RII		SII		RII		SII		RII
**Hypertension**											
2005	0.04 (0.03 to 0.05)		1.25 (1.18 to 1.33)		0.01 (0.00 to 0.03)^c^		1.08 (0.99 to 1.19)^c^		0.07 (0.05 to 0.08)		1.44 (1.31 to 1.57)
2007/2008	0.05 (0.04 to 0.06)		1.28 (1.20 to 1.37)		0.02 (0.00 to 0.04)^c^		1.10 (1.00 to 1.22)^c^		0.08 (0.06 to 0.09)		1.50 (1.38 to 1.63)
2009/2010	0.06 (0.05 to 0.08)		1.37 (1.27 to 1.47)		0.03 (0.01 to 0.05)		1.18 (1.07 to 1.30)		0.09 (0.07 to 0.11)		1.59 (1.44 to 1.75)
2011/2012	0.06 (0.05 to 0.08)		1.36 (1.26 to 1.46)		0.04 (0.01 to 0.06)		1.19 (1.07 to 1.33)		0.09 (0.07 to 0.11)		1.55 (1.41 to 1.71)
2013/2014	0.07 (0.06 to 0.09)		1.42 (1.32 to 1.53)		0.04 (0.02 to 0.07)		1.22 (1.09 to 1.37)		0.10 (0.08 to 0.12)		1.69 (1.54 to 1.86)
2015/2016	0.06 (0.05 to 0.08)		1.34 (1.26 to 1.43)		0.04 (0.02 to 0.06)		1.21 (1.10 to 1.32)		0.09 (0.07 to 0.10)		1.52 (1.39 to 1.66)
*P* for trend	.004		.01		.004		.01		.005		.002
Direction of trend	Increase		Increase		Increase		Increase		Increase		Increase
**Diabetes**											
2005	0.05 (0.04 to 0.06)	2.60 (2.28 to 2.98)		0.05 (0.03 to 0.06)		2.11 (1.76 to 2.53)		0.06 (0.05 to 0.07)		3.45 (2.84 to 4.20)	
2007/2008	0.06 (0.05 to 0.07)	2.51 (2.19 to 2.88)		0.06 (0.04 to 0.07)		2.24 (1.82 to 2.76)		0.06 (0.05 to 0.07)		2.92 (2.41 to 3.53)	
2009/2010	0.06 (0.05 to 0.07)	2.44 (2.11 to 2.81)		0.06 (0.04 to 0.07)		2.06 (1.72 to 2.46)		0.06 (0.05 to 0.08)		3.15 (2.53 to 3.91)	
2011/2012	0.06 (0.05 to 0.07)	2.43 (2.08 to 2.84)		0.06 (0.05 to 0.08)		2.26 (1.87 to 2.72)		0.06 (0.05 to 0.08)		2.67 (2.10 to 3.40)	
2013/2014	0.06 (0.05 to 0.07)	2.41 (2.11 to 2.76)		0.05 (0.04 to 0.07)		1.95 (1.63 to 2.33)		0.07 (0.06 to 0.08)		3.24 (2.71 to 3.88)	
2015/2016	0.07 (0.06 to 0.08)	2.46 (2.16 to 2.81)		0.07 (0.05 to 0.08)		2.15 (1.83 to 2.54)		0.07 (0.06 to 0.09)		2.95 (2.38 to 3.64)	
*P* for trend	.07	.75		.29		.86		.12		.75	
Direction of trend	None	None		None		None		None		None	
**Obesity**											
2005	0.05 (0.04 to 0.06)	1.37 (1.27 to 1.47)		0.00 (−0.01 to 0.02)		1.02 (0.92 to 1.13)		0.10 (0.08 to 0.12)		1.95 (1.74 to 2.18)	
2007/2008	0.06 (0.05 to 0.08)	1.43 (1.32 to 1.55)		0.02 (0.00 to 0.04)		1.11 (0.99 to 1.25)		0.10 (0.09 to 0.12)		1.91 (1.72 to 2.13)	
2009/2010	0.03 (0.01 to 0.05)	1.18 (1.08 to 1.29)		−0.01 (−0.04 to 0.01)		0.93 (0.83 to 1.04)		0.07 (0.05 to 0.10)		1.56 (1.38 to 1.77)	
2011/2012	0.03 (0.02 to 0.05)	1.19 (1.10 to 1.30)		−0.02 (−0.04 to 0.00)		0.89 (0.80 to 1.00)		0.09 (0.06 to 0.11)		1.63 (1.44 to 1.84)	
2013/2014	0.05 (0.03 to 0.06)	1.27 (1.17 to 1.38)		0.00 (−0.03 to 0.02)		0.99 (0.88 to 1.12)		0.10 (0.08 to 0.12)		1.69 (1.52 to 1.89)	
2015/2016	0.06 (0.05 to 0.08)	1.35 (1.26 to 1.45)		0.02 (0.00 to 0.04)		1.09 (0.99 to 1.21)		0.10 (0.08 to 0.12)		1.74 (1.56 to 1.93)	
*P* for trend	.90	.23		.88		>.99		.94		.06	
Direction of trend	None	None		None		None		None		None	
**Smoking**											
2005	0.15 (0.14 to 0.17)	1.95 (1.83 to 2.09)		0.16 (0.13 to 0.18)		1.85 (1.70 to 2.02)		0.15 (0.13 to 0.17)		2.09 (1.90 to 2.30)	
2007/2008	0.18 (0.16 to 0.19)	2.16 (2.01 to 2.32)		0.20 (0.17 to 0.23)		2.14 (1.95 to 2.35)		0.15 (0.13 to 0.17)		2.20 (1.99 to 2.42)	
2009/2010	0.19 (0.17 to 0.21)	2.44 (2.26 to 2.63)		0.22 (0.19 to 0.25)		2.42 (2.18 to 2.68)		0.16 (0.14 to 0.18)		2.47 (2.22 to 2.76)	
2011/2012	0.18 (0.16 to 0.19)	2.31 (2.13 to 2.49)		0.19 (0.16 to 0.22)		2.18 (1.95 to 2.43)		0.16 (0.14 to 0.18)		2.47 (2.21 to 2.77)	
2013/2014	0.18 (0.16 to 0.20)	2.52 (2.33 to 2.74)		0.21 (0.18 to 0.23)		2.43 (2.18 to 2.71)		0.15 (0.13 to 0.18)		2.66 (2.35 to 3.01)	
2015/2016	0.17 (0.16 to 0.19)	2.57 (2.37 to 2.79)		0.18 (0.15 to 0.20)		2.29 (2.06 to 2.56)		0.17 (0.15 to 0.19)		3.02 (2.67 to 3.41)	
*P* for trend	.43	<.001		.59		.01		.24		<.001	
Direction of trend	None	Increase		None		Increase		None		Increase	

Abbreviations: SII, slope index of inequality; RII, relative index of inequality.

^a^ SII and RII are adjusted for age and sex.

^b^ SII and RII are only adjusted for age.

## Discussion

The present study provides valuable evidence regarding the secular trends and geographic and socioeconomic patterns of major cardiovascular risk factors among Canadian adults between 2005 and 2016. Despite a favorable trend for current smoking, continuously increasing trends of hypertension, diabetes, and obesity were observed for both sexes. Geographically, great variability in the prevalence of these risk factors was found between health regions, although the patterns of change over time in most regions followed the national trends. In addition, persistent socioeconomic inequalities in hypertension, diabetes, and current smoking as well as obesity among women should warrant more attention.

Between 2005 and 2016, the overall tendency of hypertension, diabetes, obesity, and current smoking in Canada continued the trends of the previous decade.^6^ Despite the remarkable achievements in the control of hypertension and diabetes,^25^ the prevalence of these 2 key risk factors is still increasing. These trends may be partly due to enhanced detection rates, but modern lifestyle changes, such as physical inactivity and processed food, also play a role. Encouragingly, we noted stable changes in hypertension and diabetes among young adults as compared with a previous study.^6^

Perhaps more worrisome is the high prevalence of obesity, which has increased at a much faster rate than that during 1994 to 2005.^6^ Given that obesity accounts for a significant amount of health care expenditure in Canada^26^ and can increase the risks of hypertension and diabetes,^27^ it is urgent to galvanize more effective actions on the prevention and management of obesity. Additionally, in line with global smoking trends,^28^ the smoking rate in Canada decreased from 22.1% to 17.8% during our study period. Nevertheless, several regions still experienced an increasing trend of smoking, such as Campbellton and Nunavut. Moreover, there is a considerable variation in the prevalence of smoking across health regions from 62.4% in Nunavut to 11.0% in the Richmond Health Service Delivery Area. In fact, our data also confirmed uneven distributions and complex trends of hypertension, diabetes, and obesity across health regions. These findings are particularly useful for policy makers to inform future resource allocation and design targeted interventions.

Similar to other developed countries,^29,30,31,32,33^ we found persistent socioeconomic inequalities in hypertension, diabetes, and smoking in Canada over the past decade. This may imply that current prevention policies have not improved the disparities between socioeconomic groups, and some researchers believe that they may even exacerbate the inequalities.^34,35^ Thus, future policies should not only target the entire population but also reduce social inequalities by paying more attention to socially disadvantaged groups. Interestingly, the absolute and relative socioeconomic inequality in obesity was only evident in women, which is consistent with results in other countries, such as Portugal and Scotland.^36,37^ This gender-specific pattern may be partly due to the fact that women with higher socioeconomic status are more aware of the negative health effects of obesity, while low-income men are more inclined to engage in manual labor.^38,39^ Therefore, it would be wise to pay more attention to women with low income when making obesity prevention strategies in Canada. Notably, identifying variations across health regions and socioeconomic groups is one step in examining the barriers to reducing major cardiovascular risk factors in Canadian population. Further study is required to elucidate which interventions can improve this situation.

### Limitations

This study has limitations. First, data for hypertension, diabetes, obesity, and smoking were based on self-reports, which may lead to measurement errors and recall bias. Although measured indicators, such as measured blood pressure, were available in the Canadian Health Measures Survey (CHMS), CHMS cannot provide health information at the health region level. Additionally, given the universal health care system in Canada, the prevalence of undiagnosed hypertension and diabetes was considered very low. Previous studies have suggested that the prevalence of hypertension in CCHS was quite close to that in other data sources, including CHMS.^40^ Second, we did not examine trends of other important risk factors, such as unhealthy diet, physical inactivity, and dyslipidemia, due to lack of data or inconsistent definitions across survey cycles, although they are included in the definition of ideal cardiovascular health.^41^ Third, it is important to recognize that factors such as ethnicity, disability, immigration, and residency may also intersect with socioeconomic status to affect health equity, and thus, applying intersectional theory to understand health inequalities is an avenue for future research.^42,43^

## Conclusions

This study systematically evaluated the trends and distribution patterns of major cardiovascular risk factors among Canadian adults from 2005 to 2016. Encouraging trends were found: the prevalence of hypertension and diabetes no longer increased significantly among young people, and the prevalence of current smoking decreased among all Canadian adults. However, the continued increasing prevalence of hypertension, diabetes, and obesity is a cause for concern. Moreover, the distribution and trends of these risk factors varied widely across different health regions. Furthermore, socioeconomic inequalities in hypertension, diabetes, and current smoking, as well as obesity among women, persisted between 2005 and 2016 and even widened for hypertension and current smoking. Future interventions and policies targeted at reducing these cardiovascular risk factors in Canada should take into account the geographic and socioeconomic disparities.

## Notes

Supplement. **eTable 1. **Health Regions Combined to Ensure Historically Stable Units of Analysis **eTable 2. **Neighboring Health Regions Combined Due to Small Populations **eTable 3. **Age- and Sex-Adjusted Prevalence of Cardiovascular Risk Factors by Age Group **eTable 4. **Health Regions With Most Rapid Increase or Decline in Age- and Sex-Adjusted Prevalence of Cardiovascular Risk Factors **eFigure 1.** Health Region–Level Age- and Sex-Adjusted Prevalence of Hypertension **eFigure 2. **Health Region–Level Age- and Sex-Adjusted Prevalence of Diabetes **eFigure 3. **Health Region–Level Age- and Sex-Adjusted Prevalence of Obesity **eFigure 4. **Health Region–Level Age- and Sex-Adjusted Prevalence of Current Smoking **eFigure 5.** Age-Adjusted Prevalence of Cardiovascular Risk Factors by Income Quartile and Sex
