# Supplementary material for: Trends and Regional Variation in Prevalence of Cardiovascular Risk Factors and Association With Socioeconomic Status in Canada, 2005-2016
Source: JAMA Netw Open. 2021 Aug 19;4(8):e2121443. doi: 10.1001/jamanetworkopen.2021.21443 (PMC8377569; doi:10.1001/jamanetworkopen.2021.21443)
Supplement: Supplement. — eTable 1. Health Regions Combined to Ensure Historically Stable Units of Analysis eTable 2. Neighboring Health Regions Combined Due to Small Populations eTable 3. Age- and Sex-Adjusted Prevalence of Cardiovascular Risk Factors by Age Group eTable 4. Health Regions With Most Rapid Increase or Decline in Age- and Sex-Adjusted Prevalence of Cardiovascular Risk Factors eFigure 1. Health Region–Level Age- and Sex-Adjusted Prevalence of Hypertension eFigure 2. Health Region–Level Age- and Sex-Adjusted Prevalence of Diabetes eFigure 3. Health Region–Level Age- and Sex-Adjusted Prevalence of Obesity eFigure 4. Health Region–Level Age- and Sex-Adjusted Prevalence of Current Smoking eFigure 5. Age-Adjusted Prevalence of Cardiovascular Risk Factors by Income Quartile and Sex [file jamanetwopen-e2121443-s001.pdf]

Supplemental Online Content

Dai H, Younis A, Kong JD, Bragazzi NL, Wu J. Trends and regional variation in prevalence of cardiovascular risk factors and association with socioeconomic status in Canada, 2005-2016. *JAMA Netw Open.* 2021;4(8):e2121443. doi:10.1001/jamanetworkopen.2021.21443

eTable 1. Health Regions Combined to Ensure Historically Stable Units of Analysis

eTable 2. Neighboring Health Regions Combined Due to Small Populations

eTable 3. Age- and Sex-Adjusted Prevalence of Cardiovascular Risk Factors by Age Group

eTable 4. Health Regions With Most Rapid Increase or Decline in Age- and Sex-Adjusted Prevalence of Cardiovascular Risk Factors

eFigure 1. Health Region–Level Age- and Sex-Adjusted Prevalence of Hypertension

eFigure 2. Health Region–Level Age- and Sex-Adjusted Prevalence of Diabetes

eFigure 3. Health Region–Level Age- and Sex-Adjusted Prevalence of Obesity

eFigure 4. Health Region–Level Age- and Sex-Adjusted Prevalence of Current Smoking

eFigure 5. Age-Adjusted Prevalence of Cardiovascular Risk Factors by Income Quartile and Sex

This supplemental material has been provided by the authors to give readers additional information about their work.

© 2021 Dai H et al. *JAMA Network Open.*

eTable 1. Health Regions Combined to Ensure Historically Stable Units of Analysis

**Province**	**Group**	**Areas**	**Name of Combined areas**
Newfoundland and Labrador	1	Western Regional Integrated Health Authority (1013), Labrador-Grenfell Regional Integrated Health Authority (1014)	Western/Labrador-Grenfell Regional Integrated Health Authority
Nova Scotia	1	Zone 2 – Northern (1202), Zone 3 – Eastern (1203)	Zone – Northern/ Eastern
New Brunswick	1	Zone 2 (Saint John Area, 1302), Zone 3 (Fredericton Area, 1303)	Zone (Saint/ Fredericton Area)
Quebec	1	Région de l'Estrie (2405), Région de la Montérégie (2416)	Région l'Estrie/Montérégie
Manitoba	1	Winnipeg Regional Health Authority (4601), Northern Regional Health Authority (4604)	Winnipeg/Northern Regional Health Authority
Alberta	1	Calgary Zone (4832), Central Zone (4833)	Calgary/Central Zone
	2	Edmonton Zone (4834), North Zone (4835)	Edmonton/North Zone

© 2021 Dai H et al. *JAMA Network Open.*

eTable 2. Neighboring Health Regions Combined Due to Small Populations

**Province**	**Group**	**Areas**	**Name of Combined areas**
Saskatchewan	1	Mamawetan Churchill River Regional Health Authority (4711), Keewatin Yatthé Regional Health Authority (4712), and Athabasca Health Authority (4713)	Mamawetan/Keewatin/Athabasca Regional Health Authorities

© 2021 Dai H et al. *JAMA Network Open.*

eTable 3. Age- and Sex-Adjusted Prevalence of Cardiovascular Risk Factors by Age Group[a]

Age groups, years	2005	2007/08	2009/10	2011/12	2013/14	2015/16	p value	Direction of trend
**Hypertension**								
Both sexes								
20~39	3.2 (3.0-3.5)	3.3 (3.1-3.6)	3.1 (2.9-3.4)	3.2 (2.9-3.5)	3.2 (2.9-3.5)	3.7 (3.4-4.1)	0.097	—
40~59	16.8 (16.2-17.3)	17.5 (16.9-18.1)	17.7 (17.0-18.4)	17.3 (16.6-18.0)	17.5 (16.7-18.2)	17.8 (17.1-18.5)	0.096	—
60~79	43.3 (42.5-44.1)	45.1 (44.3-45.9)	46.5 (45.6-47.3)	46.9 (46.0-47.7)	45.7 (44.8-46.5)	45.9 (45.0-46.7)	<0.001	Increase
≥80	53.3 (51.7-55.0)	56.8 (55.1-58.6)	56.8 (54.9-58.7)	57.7 (56.1-59.2)	57.5 (55.9-59.2)	62.9 (61.1-64.7)	<0.001	Increase
Men								
20~39	3.8 (3.3-4.2)	3.9 (3.5-4.4)	3.9 (3.4-4.3)	3.9 (3.4-4.4)	4.0 (3.5-4.5)	4.5 (3.9-5.0)	0.071	—
40~59	17.5 (16.7-18.3)	18.4 (17.5-19.3)	19.4 (18.3-20.5)	18.9 (17.9-20.0)	20.4 (19.2-21.5)	20.4 (19.4-21.5)	<0.001	Increase
60~79	41.3 (40.1-42.5)	43.4 (42.2-44.6)	45.0 (43.7-46.3)	47.0 (45.6-48.3)	46.8 (45.6-48.0)	47.5 (46.3-48.8)	<0.001	Increase
≥80	43.6 (40.8-46.3)	50.9 (48.2-53.6)	49.5 (46.5-52.4)	51.4 (48.8-54.0)	53.9 (51.3-56.5)	56.7 (53.5-59.8)	<0.001	Increase
Women								
20~39	2.7 (2.4-3.0)	2.8 (2.4-3.1)	2.4 (2.1-2.7)	2.6 (2.2-3.0)	2.5 (2.1-2.8)	3.0 (2.5-3.5)	0.678	—
40~59	16.0 (15.3-16.8)	16.6 (15.8-17.5)	16.1 (15.1-17.1)	15.8 (14.9-16.7)	14.7 (13.8-15.6)	15.3 (14.4-16.2)	0.003	Decrease
60~79	45.0 (43.9-46.1)	46.7 (45.5-47.8)	47.7 (46.6-48.8)	46.8 (45.6-47.9)	44.6 (43.5-45.7)	44.3 (43.1-45.5)	0.021	Decrease
≥80	58.7 (56.6-60.8)	60.1 (57.8-62.4)	60.9 (58.4-63.4)	61.1 (58.8-63.3)	59.5 (57.4-61.6)	66.3 (64.2-68.5)	<0.001	Increase
**Diabetes**								
Both sexes								
20~39	1.1 (1.0-1.3)	1.3 (1.1-1.5)	1.3 (1.1-1.5)	1.1 (1.0-1.3)	1.2 (1.0-1.4)	1.4 (1.2-1.6)	0.240	—
40~59	5.0 (4.7-5.3)	6.0 (5.7-6.4)	5.9 (5.5-6.3)	5.9 (5.4-6.3)	6.2 (5.7-6.6)	6.5 (6.1-7.0)	<0.001	Increase
60~79	13.7 (13.1-14.2)	15.4 (14.8-16.1)	16.0 (15.4-16.6)	16.6 (15.9-17.4)	16.7 (16.0-17.3)	16.4 (15.7-17.1)	<0.001	Increase
≥80	13.4 (12.3-14.5)	15.9 (14.5-17.3)	18.6 (17.0-20.3)	16.5 (15.1-17.9)	16.6 (15.2-17.9)	17.4 (16.0-18.8)	<0.001	Increase

© 2021 Dai H et al. *JAMA Network Open*.

Men								
20~39	1.0 (0.8-1.2)	1.2 (1.0-1.5)	1.3 (1.0-1.7)	1.0 (0.8-1.3)	1.1 (0.8-1.4)	1.4 (1.1-1.7)	0.345	—
40~59	5.6 (5.1-6.1)	6.9 (6.3-7.5)	7.0 (6.3-7.7)	6.1 (5.5-6.8)	7.4 (6.6-8.1)	7.6 (6.9-8.3)	<0.001	Increase
60~79	16.1 (15.2-17.1)	17.6 (16.6-18.5)	19.1 (18.1-20.2)	19.4 (18.3-20.5)	19.4 (18.3-20.4)	18.8 (17.7-19.8)	<0.001	Increase
≥80	15.3 (13.2-17.3)	18.1 (15.7-20.4)	22.3 (19.8-24.8)	19.6 (17.2-21.9)	17.9 (15.9-19.8)	19.9 (17.4-22.4)	0.031	Increase
Women								
20~39	1.2 (1.0-1.4)	1.3 (1.1-1.6)	1.3 (1.1-1.5)	1.2 (1.0-1.5)	1.3 (1.0-1.6)	1.4 (1.1-1.6)	0.475	—
40~59	4.4 (4.0-4.8)	5.2 (4.7-5.7)	4.8 (4.3-5.3)	5.6 (5.0-6.2)	5.0 (4.5-5.5)	5.5 (4.9-6.1)	0.007	Increase
60~79	11.5 (10.8-12.2)	13.5 (12.6-14.3)	13.2 (12.4-14.0)	14.2 (13.3-15.0)	14.3 (13.5-15.1)	14.2 (13.3-15.2)	<0.001	Increase
≥80	12.3 (11.0-13.7)	14.6 (13.0-16.3)	16.6 (14.6-18.7)	14.8 (13.1-16.5)	15.8 (14.1-17.6)	16.0 (14.5-17.6)	0.001	Increase
**Obesity**								
Both sexes								
20~39	13.0 (12.5-13.4)	14.4 (13.8-14.9)	14.6 (14.0-15.3)	14.8 (14.1-15.6)	16.5 (15.8-17.3)	16.3 (15.6-16.9)	<0.001	Increase
40~59	18.5 (17.9-19.1)	19.1 (18.4-19.7)	20.8 (20.0-21.5)	21.4 (20.6-22.2)	22.1 (21.3-22.9)	23.3 (22.5-24.0)	<0.001	Increase
60~79	18.1 (17.4-18.7)	20.2 (19.5-20.8)	20.7 (20.0-21.4)	21.1 (20.3-21.8)	21.7 (21.0-22.4)	22.6 (21.8-23.3)	<0.001	Increase
≥80	10.3 (9.1-11.4)	11.2 (10.0-12.5)	11.8 (10.7-13.0)	11.0 (10.0-12.1)	13.4 (12.1-14.7)	12.1 (10.9-13.3)	0.003	Increase
Men								
20~39	14.5 (13.8-15.2)	15.7 (14.9-16.5)	16.1 (15.1-17.0)	15.2 (14.2-16.2)	18.5 (17.3-19.6)	17.4 (16.4-18.4)	<0.001	Increase
40~59	19.9 (19.0-20.9)	20.6 (19.6-21.6)	23.3 (22.2-24.4)	23.6 (22.5-24.7)	24.3 (23.0-25.6)	25.4 (24.3-26.5)	<0.001	Increase
60~79	18.2 (17.2-19.1)	20.4 (19.4-21.4)	20.3 (19.3-21.4)	21.4 (20.2-22.6)	21.9 (20.9-22.9)	23.7 (22.6-24.8)	<0.001	Increase
≥80	8.9 (7.3-10.6)	9.2 (7.6-10.9)	10.7 (9.0-12.5)	10.1 (8.5-11.8)	11.9 (10.1-13.7)	12.3 (10.4-14.2)	0.001	Increase
Women								
20~39	11.5 (10.9-12.1)	13.1 (12.4-13.8)	13.2 (12.3-14.1)	14.5 (13.6-15.4)	14.6 (13.7-15.5)	15.1 (14.2-16.1)	<0.001	Increase
40~59	17.2 (16.4-17.9)	17.6 (16.7-18.4)	18.3 (17.4-19.3)	19.3 (18.2-20.4)	20.0 (19.0-21.0)	21.2 (20.2-22.2)	<0.001	Increase
60~79	17.9 (17.0-18.8)	19.9 (19.0-20.9)	21.0 (20.1-22.0)	20.8 (19.9-21.7)	21.5 (20.7-22.4)	21.5 (20.5-22.5)	<0.001	Increase

© 2021 Dai H et al. *JAMA Network Open*.

≥80	11.0 (9.6-12.4)	12.3 (10.6-14.0)	12.4 (10.8-14.0)	11.5 (10.1-13.0)	14.3 (12.6-16.0)	12.0 (10.4-13.6)	0.154	—
**Current smoking**								
Both sexes								
20~39	26.9 (26.2-27.6)	26.5 (25.7-27.2)	24.2 (23.4-24.9)	24.3 (23.4-25.1)	22.5 (21.7-23.3)	20.3 (19.6-21.0)	<0.001	Decrease
40~59	24.0 (23.4-24.7)	24.6 (23.9-25.4)	23.5 (22.7-24.2)	23.6 (22.8-24.5)	21.3 (20.5-22.2)	19.9 (19.2-20.6)	<0.001	Decrease
60~79	13.7 (13.1-14.2)	13.6 (13.0-14.1)	13.6 (13.0-14.1)	12.7 (12.1-13.2)	12.7 (12.2-13.3)	12.5 (11.9-13.1)	<0.001	Decrease
≥80	5.8 (4.9-6.7)	5.0 (4.3-5.7)	4.9 (4.2-5.7)	4.7 (4.0-5.5)	4.5 (3.9-5.2)	3.8 (3.1-4.4)	<0.001	Decrease
Men								
20~39	30.5 (29.5-31.6)	30.4 (29.3-31.5)	28.0 (26.8-29.3)	27.7 (26.5-28.9)	27.2 (25.9-28.5)	24.1 (22.9-25.3)	<0.001	Decrease
40~59	25.5 (24.5-26.5)	27.2 (26.1-28.3)	26.4 (25.2-27.5)	26.7 (25.3-28.0)	24.4 (23.1-25.7)	22.4 (21.4-23.5)	<0.001	Decrease
60~79	14.5 (13.6-15.3)	14.7 (13.8-15.6)	15.0 (14.2-15.8)	13.6 (12.7-14.5)	14.0 (13.1-14.9)	14.0 (13.1-14.8)	0.123	—
≥80	6.6 (4.9-8.3)	5.3 (4.2-6.4)	5.5 (4.2-6.9)	5.5 (4.2-6.8)	5.5 (4.3-6.7)	3.1 (2.3-4.0)	0.001	Decrease
Women								
20~39	23.3 (22.5-24.2)	22.6 (21.7-23.5)	20.5 (19.5-21.4)	20.9 (19.7-22.0)	17.9 (17.0-18.8)	16.6 (15.7-17.6)	<0.001	Decrease
40~59	22.6 (21.7-23.5)	22.1 (21.2-23.0)	20.6 (19.6-21.6)	20.7 (19.7-21.7)	18.4 (17.3-19.4)	17.5 (16.6-18.4)	<0.001	Decrease
60~79	12.9 (12.3-13.6)	12.5 (11.8-13.3)	12.2 (11.5-12.9)	11.8 (11.1-12.5)	11.6 (10.9-12.2)	11.1 (10.4-11.9)	<0.001	Decrease
≥80	5.3 (4.4-6.3)	4.8 (4.0-5.6)	4.6 (3.7-5.5)	4.3 (3.4-5.3)	4.0 (3.2-4.8)	4.2 (3.2-5.1)	0.025	Decrease
**≥2 risk factors**								
Both sexes								
20~39	5.5 (5.2-5.8)	6.3 (5.9-6.6)	6.0 (5.5-6.4)	6.0 (5.5-6.5)	6.2 (5.8-6.6)	6.2 (5.8-6.7)	0.042	Increase
40~59	13.2 (12.7-13.7)	14.2 (13.6-14.8)	14.9 (14.3-15.6)	15.1 (14.4-15.8)	14.8 (14.1-15.5)	15.6 (14.9-16.2)	<0.001	Increase
60~79	22.1 (21.4-22.8)	24.3 (23.6-25.1)	25.6 (24.9-26.3)	25.8 (24.9-26.7)	26.4 (25.7-27.1)	26.0 (25.2-26.8)	<0.001	Increase
≥80	17.0 (15.7-18.4)	18.4 (16.9-19.9)	21.0 (19.2-22.9)	19.2 (17.7-20.7)	20.3 (18.8-21.8)	22.9 (21.3-24.5)	<0.001	Increase
Men								

© 2021 Dai H et al. *JAMA Network Open*.

20~39	6.2 (5.7-6.7)	7.1 (6.5-7.7)	6.8 (6.2-7.4)	6.2 (5.6-6.8)	7.4 (6.6-8.2)	7.2 (6.5-7.9)	0.036	Increase
40~59	14.1 (13.3-14.8)	15.9 (15.0-16.8)	17.3 (16.2-18.3)	17.2 (16.0-18.3)	17.7 (16.6-18.8)	18.3 (17.2-19.3)	<0.001	Increase
60~79	22.8 (21.7-23.8)	25.2 (24.2-26.3)	26.7 (25.6-27.9)	27.5 (26.1-28.9)	28.3 (27.2-29.5)	28.3 (27.1-29.5)	<0.001	Increase
≥80	15.6 (13.5-17.6)	17.0 (14.5-19.6)	20.3 (17.7-22.8)	18.9 (16.5-21.4)	20.3 (18.1-22.5)	23.9 (21.0-26.8)	<0.001	Increase
Women								
20~39	4.9 (4.5-5.3)	5.5 (5.0-5.9)	5.1 (4.6-5.7)	5.7 (5.0-6.5)	5.0 (4.5-5.5)	5.2 (4.6-5.8)	0.676	—
40~59	12.3 (11.7-13.0)	12.6 (11.9-13.3)	12.6 (11.8-13.5)	13.1 (12.2-14.0)	11.9 (11.2-12.7)	13.0 (12.1-13.8)	0.626	—
60~79	21.5 (20.6-22.4)	23.5 (22.5-24.5)	24.5 (23.5-25.5)	24.2 (23.2-25.3)	24.6 (23.7-25.6)	23.8 (22.7-24.8)	0.001	Increase
≥80	17.8 (16.1-19.6)	19.1 (17.2-21.0)	21.5 (19.1-23.8)	19.3 (17.5-21.2)	20.3 (18.4-22.2)	22.3 (20.4-24.2)	0.002	Increase

Numbers in the table are percentage (95% confidence interval).

[a] Because 10-year interval of age is used to calculate age- and sex-adjusted prevalence of cardiovascular risk factors in this study, age comparisons with 20-year interval in the Table are still adjusted for age. Sex comparisons are not adjusted for sex.

© 2021 Dai H et al. *JAMA Network Open*.

eTable 4. Health regions With Most Rapid Increase or Decline in Age- and Sex-Adjusted Prevalence of Cardiovascular Risk Factors

	Most rapid increase		Most rapid decrease	
	**Health regions**	**Percent change**	**Health regions**	**Percent change**
**Hypertension**				
	Kelsey Trail Regional Health Authority	56.8%	Région de l'Outaouais	-22.9%
	East Kootenay Health Service Delivery Area	43.0%	Edmundston Area	-18.1%
	Prince Albert Parkland Regional Health Authority	30.9%	North Shore/Coast Garibaldi Health Service Delivery Area	-13.4%
	Cypress Regional Health Authority	30.8%	Interlake‑Eastern Regional Health Authority	-10.6%
	Sun Country Regional Health Authority	30.3%	Vancouver Health Service Delivery Area	-10.0%
**Diabetes**				
	North Vancouver Island Health Service Delivery Area	95.2%	Kootenay‑Boundary Health Service Delivery Area	-44.9%
	City of Toronto Health Unit	90.5%	North Bay Parry Sound District Health Unit	-26.1%
	Halton Regional Health Unit	86.9%	Fraser North Health Service Delivery Area	-22.5%
	Five Hills Regional Health Authority	74.4%	The District of Algoma Health Unit	-22.4%
	Lambton Health Unit	71.9%	Okanagan Health Service Delivery Area	-21.8%
**Obesity**				
	Leeds, Grenville and Lanark District Health Unit	93.8%	Richmond Health Service Delivery Area	-36.2%
	Kingston, Frontenac and Lennox and Addington Health Unit	81.2%	Vancouver Health Service Delivery Area	-23.0%
	Haliburton, Kawartha, Pine Ridge District Health Unit	80.6%	Central Vancouver Island Health Service Delivery Area	-16.2%
	Région de l'Abitibi‑Témiscamingue	75.6%	Nunavut	-10.2%
	Région de la Côte‑Nord	65.5%	Heartland Regional Health Authority	-5.5%
**Current smoking**				
	Campbellton area	86.9%	Cypress Regional Health	-46.4%

© 2021 Dai H et al. *JAMA Network Open.*

			Authority	
	Nunavut	35.5%	Peel Regional Health Unit	-38.5%
	The District of Algoma Health Unit	33.9%	Moncton Area	-38.2%
	Mamawetan/Keewatin/Athabasca Regional Health Authorities	18.1%	Haldimand-Norfolk Health Unit	-36.6%
	Western/Labrador-Grenfell Regional Integrated Health Authority	17.3%	Région de Laval	-35.9%
**≥2 risk factors**				
	Haliburton, Kawartha, Pine Ridge District Health Unit	80.9%	Vancouver Health Service Delivery Area	-17.6%
	City of Toronto Health Unit	55.7%	Prairie North Regional Health Authority	-13.0%
	The District of Algoma Health Unit	53.5%	South Vancouver Island Health Service Delivery Area	-10.0%
	Eastern Regional Integrated Health Authority	44.9%	Central Vancouver Island Health Service Delivery Area	-8.3%
	Western/Labrador-Grenfell Regional Integrated Health Authority	44.8%	Région de l'Outaouais	-7.6%

© 2021 Dai H et al. *JAMA Network Open.*

eFigure 1. Health Region–Level Age- and Sex-Adjusted Prevalence of Hypertension

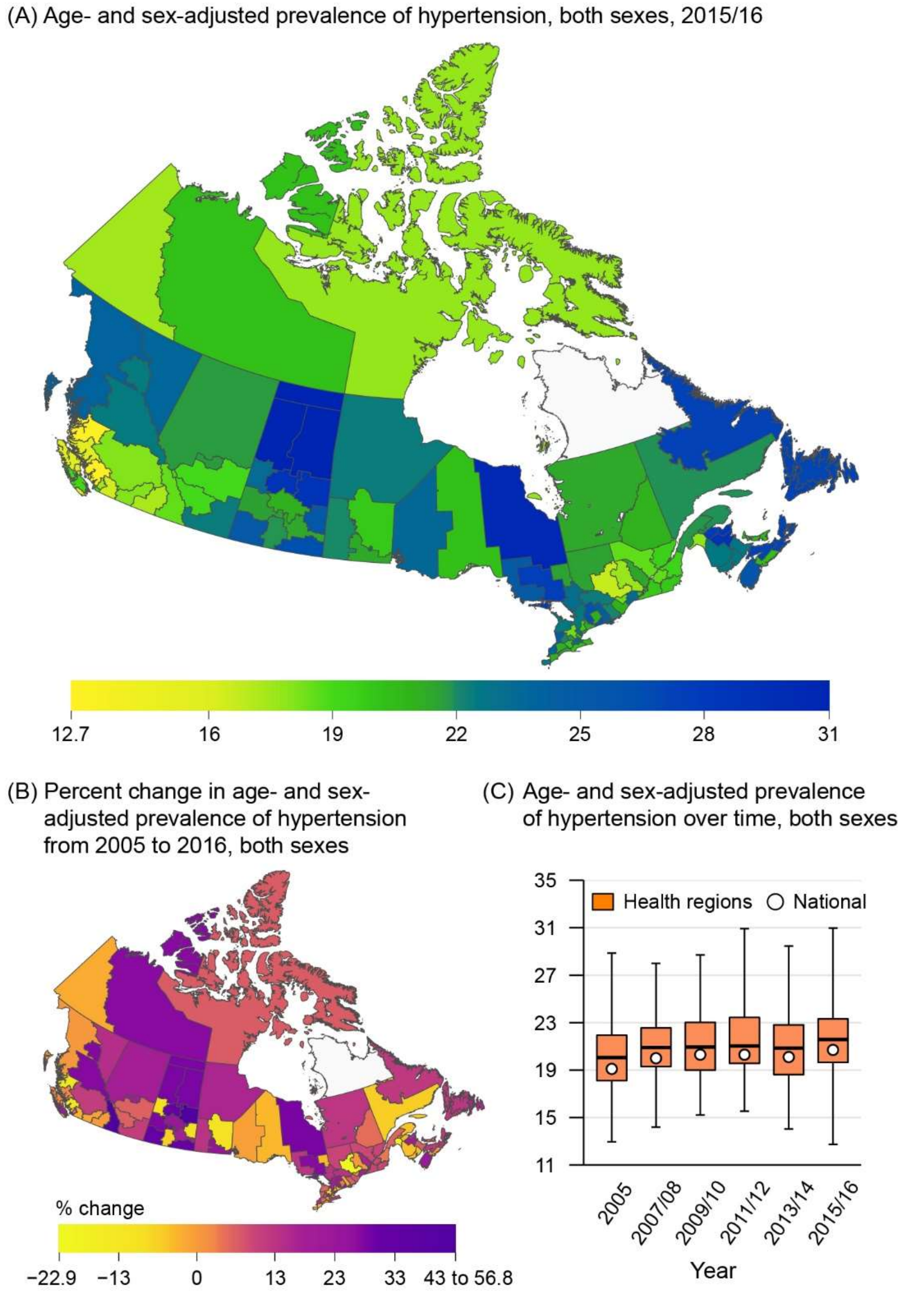

A) Age- and sex-adjusted prevalence of hypertension for both sexes combined in 2015/16. B) Percent change in the age- and sex-adjusted prevalence of hypertension for both sexes combined from 2005 to 2016. In A) and B), two health regions (i.e. Région du Nunavik, and Région des Terres Cries de la Baie James) were filled with blank because of missing data. C) Age- and sex-adjusted prevalence of hypertension in each survey cycle. The bottom border, middle line, and top border of the boxes indicate the 25th, 50th, and 75th percentiles, respectively, across all health regions; the whiskers indicate the full range across all health regions; and the circles indicate the national-level prevalence rate.

© 2021 Dai H et al. *JAMA Network Open.*

eFigure 2. Health Region–Level Age- and Sex-Adjusted Prevalence of Diabetes

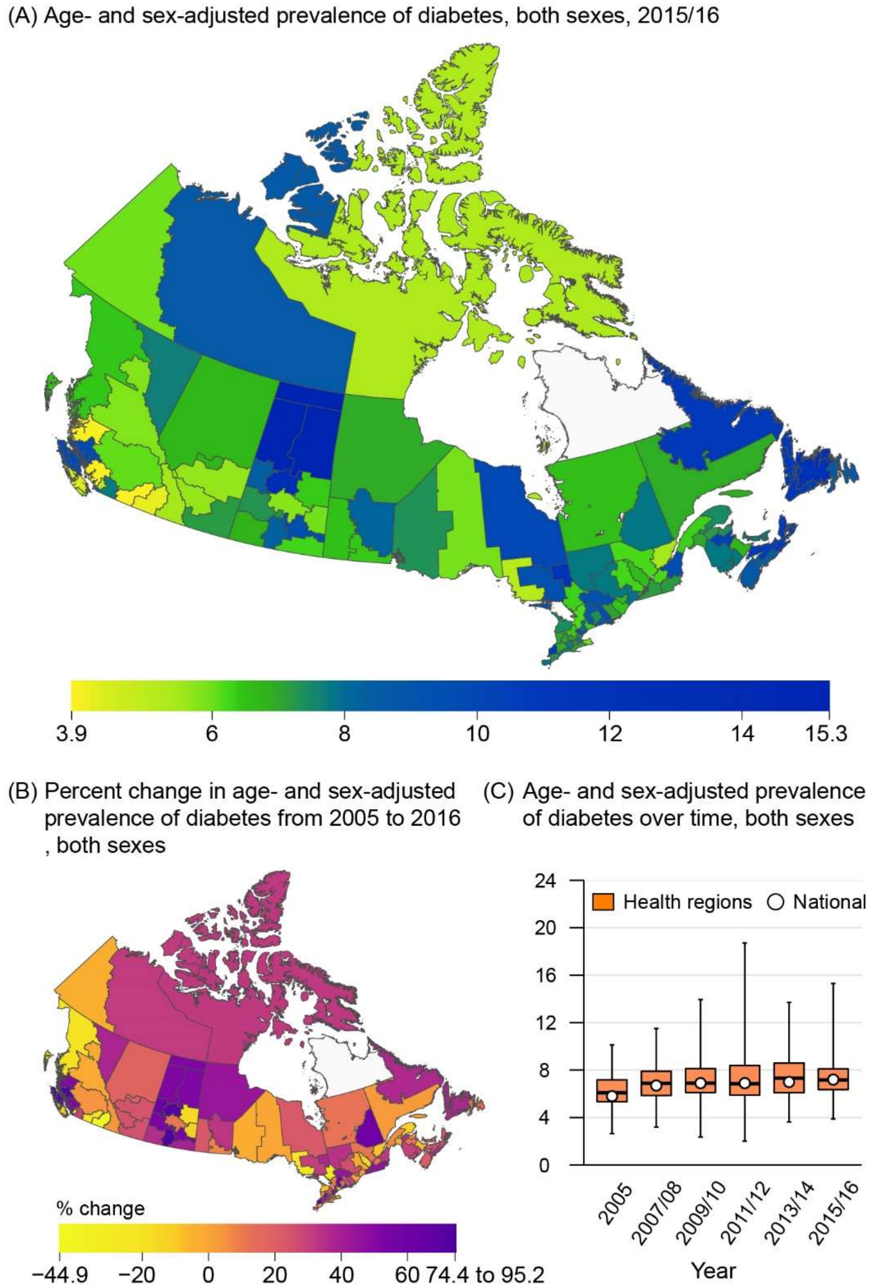

A) Age- and sex-adjusted prevalence of diabetes for both sexes combined in 2015/16. B) Percent change in the age- and sex-adjusted prevalence of diabetes for both sexes combined from 2005 to 2016. In A) and B), two health regions (i.e. Région du Nunavik, and Région des Terres Cries de la Baie James) were filled with blank because of missing data. C) Age- and sex-adjusted prevalence of diabetes in each survey cycle. The bottom border, middle line, and top border of the boxes indicate the 25th, 50th, and 75th percentiles, respectively, across all health regions; the whiskers indicate the full range across all health regions; and the circles indicate the national-level prevalence rate.

© 2021 Dai H et al. *JAMA Network Open.*

eFigure 3. Health Region–Level Age- and Sex-Adjusted Prevalence of Obesity

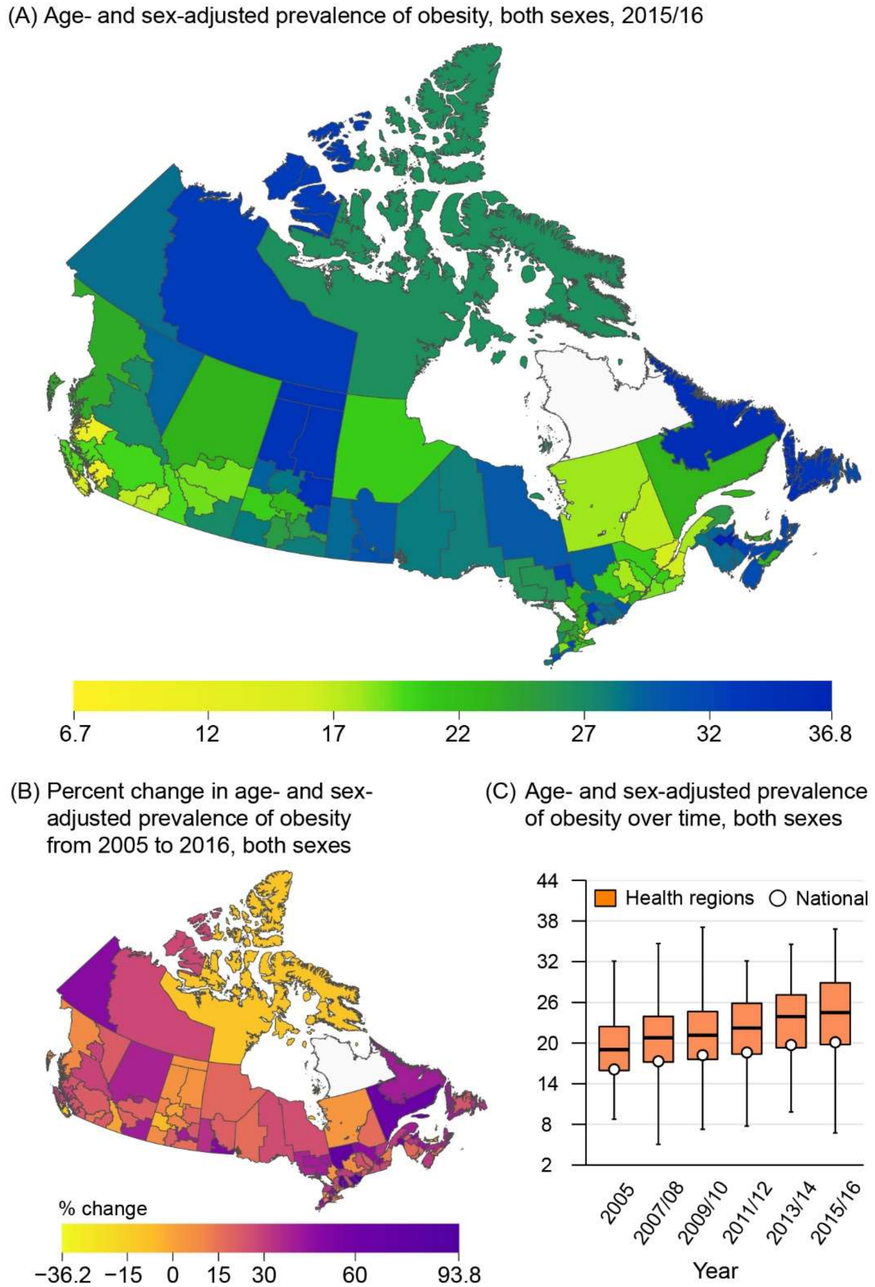

A) Age- and sex-adjusted prevalence of obesity for both sexes combined in 2015/16. B) Percent change in the age- and sex-adjusted prevalence of obesity for both sexes combined from 2005 to 2016. In A) and B), two health regions (i.e. Région du Nunavik, and Région des Terres Cries de la Baie James) were filled with blank because of missing data. C) Age- and sex-adjusted prevalence of obesity in each survey cycle. The bottom border, middle line, and top border of the boxes indicate the 25th, 50th, and 75th percentiles, respectively, across all health regions; the whiskers indicate the full range across all health regions; and the circles indicate the national-level prevalence rate.

© 2021 Dai H et al. *JAMA Network Open.*

eFigure 4. Health Region–Level Age- and Sex-Adjusted Prevalence of Current Smoking

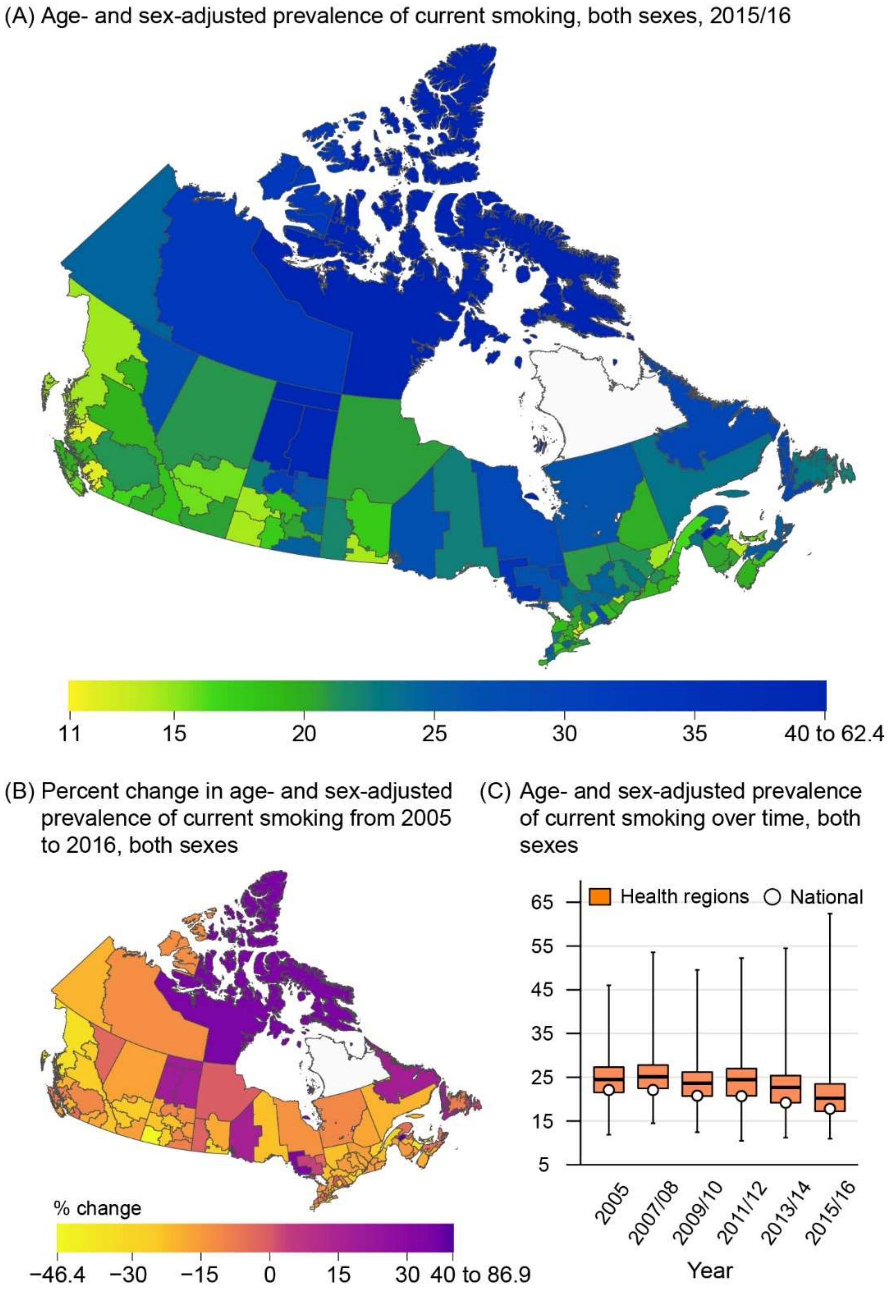

A) Age- and sex-adjusted prevalence of current smoking for both sexes combined in 2015/16. B) Percent change in the age- and sex-adjusted prevalence of current smoking for both sexes combined from 2005 to 2016. In A) and B), two health regions (i.e. Région du Nunavik, and Région des Terres Cries de la Baie James) were filled with blank because of missing data. C) Age- and sex-adjusted prevalence of current smoking in each survey cycle. The bottom border, middle line, and top border of the boxes indicate the 25th, 50th, and 75th percentiles, respectively, across all health regions; the whiskers indicate the full range across all health regions; and the circles indicate the national-level prevalence rate.

© 2021 Dai H et al. *JAMA Network Open.*

eFigure 5. Age-Adjusted Prevalence of Cardiovascular Risk Factors by Income Quartile and Sex

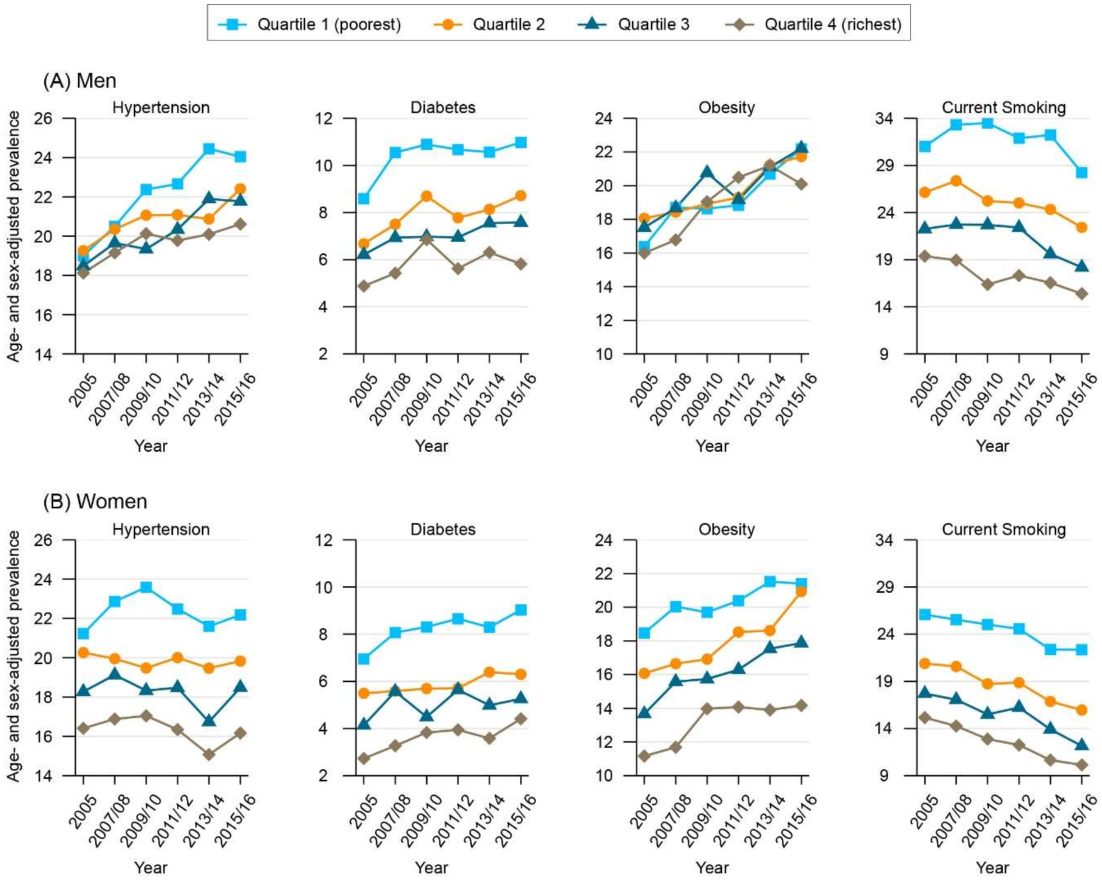

Sex comparisons are only adjusted for age.

© 2021 Dai H et al. *JAMA Network Open.*
